# Adropin Predicts Chronic Kidney Disease in Type 2 Diabetes Mellitus Patients with Chronic Heart Failure

**DOI:** 10.3390/jcm12062231

**Published:** 2023-03-13

**Authors:** Tetiana A. Berezina, Zeljko Obradovic, Elke Boxhammer, Alexander A. Berezin, Michael Lichtenauer, Alexander E. Berezin

**Affiliations:** 1 Department of Nephrology, “Vita Center”, 3, Sedov Str., 69000 Zaporozhye, Ukraine; 2 Klinik Barmelweid, Department of Psychosomatic Medicine and Psychotherapy, 5017 Barmelweid, Switzerland; 3 Department of Internal Medicine II, Division of Cardiology, Paracelsus Medical University, Strubergasse 21, 5020 Salzburg, Austria; 4 Department of Internal Medicine, Zaporozhye Medical Academy of Postgraduate Education, 20, Vinter Av., 69096 Zaporozhye, Ukraine; 5 Department of Internal Medicine, Zaporozhye State Medical University, 26, Mayakovsky Av., 69035 Zaporozhye, Ukraine

**Keywords:** type 2 diabetes mellitus, heart failure, chronic kidney disease, adropin, biomarkers, prediction

## Abstract

Adropin is a multifunctional secreted protein, which is involved in the metabolic modulation of the heart-brain-kidney axis in heart failure (HF). The aim of the study was to detect the plausible predictive value of serum levels of adropin for chronic kidney disease (CKD) grades 1–3 in type 2 diabetes mellitus (T2DM) patients with chronic HF. We enrolled 417 T2DM individuals with chronic HF and subdivided them into two groups depending on the presence of CKD. The control group was composed of 25 healthy individuals and 30 T2DM patients without HF and CKD. All eligible patients underwent an ultrasound examination. Adropin was detected by ELISA in blood samples at the study baseline. We found that adropin levels in T2DM patients without HF and CKD were significantly lower than in healthy volunteers, but they were higher than in T2DM patients with known HF. The optimal cut-off point for adropin levels was 2.3 ng/mL (area under the curve [AUC] = 0.86; 95% CI = 0.78–0.95; sensitivity = 81.3%, specificity = 77.4%). The multivariate logistic regression adjusted for albuminuria/proteinuria showed that serum levels of adropin <2.30 ng/mL (OR = 1.55; *p* = 0.001) independently predicted CKD. Conclusions: Low levels of adropin in T2DM patients with chronic CH seem to be an independent predictor of CKD at stages 1–3.

## 1. Introduction

Patients with type 2 diabetes mellitus (T2DM) and chronic heart failure (HF) are at increased risk of newly developing chronic kidney disease (CKD), which interferes with mortality and quality of life [1]. On the other hand, T2DM and/or HF are common comorbidities in patients with pre-existent CKD [2,3]. These conditions are closely overlapped in conventional cardiovascular (CV) and unique kidney-specific (anaemia, malnutrition, altered bone mineral metabolism, etc.) risk factors and strongly pathophysiologically interrelated by the complex relationship between myocardial, vascular, and renal injury [4]. These risk factors are found to be sufficient in contributing to the decline of kidney function, which is frequently noticed in patients with any phenotype of HF [5]. Indeed, there are a large number of common pathways, such as systemic and microvascular inflammation, altered cellular immune reactions, oxidative stress and mitochondrial dysfunction, neurohormonal activation, skeletal muscle and adipose tissue dysfunction, which are sustained by glucose and lipid toxicity, impaired nutritional status, altered acid-base, and fluid condition [4,5].

Despite the implementation of conventional management, the incidence rate and, consequently, the prevalence of CKD among HF patients continue to increase [6]. This growth is provoked by the age and increased life span of HF patients as well as a signature of comorbidities, which in particular include T2DM as a global factor that has reached pandemic levels worldwide [7]. The presence of CKD often intervenes in the decision to initiate and maintain life-saving HF therapies among T2DM patients with HF [8]. In addition to that, the synthesis, clearance, peak diagnostic values, and predictive capabilities of the majority of conventional cardiac biomarkers, including natriuretic peptides, are affected by CKD [9]. Yet, new management of HF with sodium-glucose cotransporter 2 (SGLT2) inhibitors was found to be effective in improving clinical outcomes regardless of the circulating levels of N-terminal pro-B-type natriuretic peptide (NT-proBNP) [10,11]. Interestingly, kidney injury biomarkers are not validated to change HF management, whereas continuous monitoring of NPs in HF patients with concomitant T2DM and CKD has limited evidence of its efficacy [12]. In this context, the discovery of novel biomarker-guided approaches to predict CKD and its evolution among HF patients with T2DM with any concentrations of natriuretic peptides (NPs) seems to be promising.

Adropin is a multifunctional peptide, which, being primarily secreted by the liver and brain, modulates the metabolic homeostasis of the heart, vasculature, kidney, and skeletal muscles in connection with nutrition status [13]. Adropin exerts its biological effects through binding with three distinct membrane receptors, which seem to be responsible for various modulations of target tissue metabolism. In fact, the Nb-3/Notch signalling pathway is suppressed by adropin, which thereby promotes a central inhibitory effect on water deprivation-induced drinking [14]. Through a canonical cascade including the G-coupled protein receptor 19 (GPR19)–mitogen-activated protein kinase (MAPK)–pyruvate dehydrogenase lipoamide kinase isozyme 4 (PDK4) pathway, adropin downregulates the expression of PDK-4 and consequently mediates metabolic homeostasis of cardiac cells [15]. Finally, favourable effects of adropin on vascular structure and function are mediated by vascular endothelial growth factor (VEGF) via binding with VEGF receptor 2 (VEGFR2) [16]. Yet, adropin may activate the glucose transporter 4 receptor through its Akt phosphorylation and improve glucose metabolism [16]. There is strong evidence regarding the fact that adropin is able to inhibit inflammation by suppressing the production of several pro-inflammatory cytokines (tumour necrosis factor alpha, C-reactive protein, and interleukin-6), improve cardiac function and coronary blood flow, reduce the levels of serum triglycerides, total cholesterol, and low-density lipoprotein cholesterol, and increase the level of high-density lipoprotein cholesterol [17,18,19,20].

The clinical significance of adropin levels in different patient populations remains controversial. There is numerous evidence that overweight/obese/T2DM patients had lower adropin levels than healthy volunteers and that a decreased adropin level was associated with a risk of renal dysfunction in patients with T2DM [21,22,23,24]. Another study reported that elevated serum levels of adropin correlated with a low risk of carotid atherosclerosis in T2DM patients [25]. Yet, elevated serum adropin levels after treatment with sitagliptin or SGLT2 inhibitors were strongly associated with improvements in fasting blood glucose, glycosylated haemoglobin (HbA1c), insulin sensitivity, and NP levels [26,27]. Along with it, aerobic exercise training was able to increase plasma levels of adropin in connection with blood pressure reduction by increasing nitric oxide production and bioavailability [28]. On the contrary, in patients with cardiac dysfunction, the serum levels of adropin increased significantly according to the New York Heart Association (NYHA) class of HF and demonstrated a tendency to decrease during treatment with hydralazine combined with sodium nitroprusside and SGLT2 inhibitors [27,29,30,31]. Although low levels of adropin predicted CKD in T2DM and high levels of adropin were associated with HF, there is no certain evidence that adropin has a discriminative value for CKD in HF patients with T2DM [32]. The aim of the study was to detect the plausible predictive value of serum levels of adropin for CKD 1–3 grades in T2DM patients with chronic HF.

## 2. Materials and Methods

### 2.1. Research Object and Patient Characteristics

**Figure 1:**
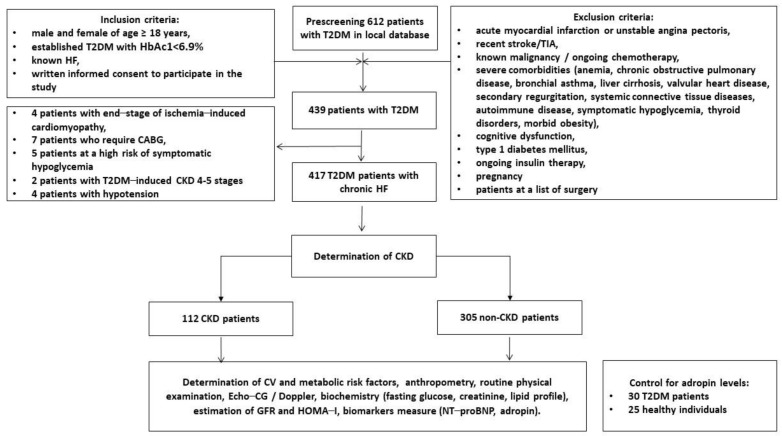
Flow chart of the study design. Abbreviations: CABG, coronary artery bypass grafting; CV, cardiovascular; CKD, chronic kidney disease; GFR, glomerular filtration rate; HF, heart failure; HbA1c, glycated haemoglobin; HOMA–IR, Homeostatic Assessment Model of Insulin Resistance; N–terminal brain natriuretic pro-peptide; T2DM, type 2 diabetes mellitus; TIA, transient ischemic attack.

This is a clinical cohort study in which patients with T2DM were enrolled from the local database of the private hospital “Vita-Center” (Zaporozhye, Ukraine). A total of 612 patients with T2DM were selected according to inclusion and exclusion criteria, which are indicated in Figure 1. We excluded patients who had end-stage target organ disease (4 patients with end-stage ischemia-induced cardiomyopathy, 4 patients with CKD 4–5 stages), severe symptoms of hypoglycemia (5 patients), or hypotension (4 patients), and those who were listed as candidates for surgical procedures (7 patients who require CABG). Finally, we enrolled 417 individuals with T2DM who had chronic HF and subdivided them into two groups depending on the presence of CKD 1–3 grades (estimated GFR > 30 mL/min/1.73 m^2^). At the same time, 25 healthy individuals and 30 patients with T2DM without HF and CKD were included in the study as controls.

The diagnosis of CKD was established according to the definition of CKD in the Kidney Disease Improving Global Outcomes (KDIGO) Consensus Report [33]. Albuminuria/proteinuria were defined as urine albumin to creatinine ratio = 30–300 mg/g and urine total protein to creatinine ratio > 300 mg/g [34]. T2DM and HF were established according to conventional clinical recommendations [35,36]. We determined HF with reduced EF (HFrEF) as HF with a left ventricular ejection fraction (LVEF) of ≤40%; HF with mildly reduced EF (HFmrEF) as HF with an LVEF of 41–49%; and HF with preserved EF (HFpEF) as HF with an LVEF of ≥50% [36]. The European Society of Cardiology (ESC) clinical guidelines were used to determine concomitant diseases and CV risk factors, such as hypertension [37], dyslipidemia [38], and coronary artery disease/chronic coronary syndrome [39].

### 2.2. Determination of Anthropometric Parameters, Co-Morbidities, and Concomitant Diseases

Standard anthropometric features including height (cm), weight (kg), waist circumference (cm), hip-to-waist ratio (WHR), body mass index (BMI), and body surface area (BSA) were measured according to current recommendations [40].

### 2.3. Hemodynamic Features

All eligible patients underwent B-mode echocardiography and impulse/tissue Doppler examinations obtained with a commercially available diagnostic system, Vivid T8 (“GE Medical Systems”, Freiburg, Germany), by a blinded ultrasonographer in compliance with current guidelines [41]. Left ventricular (LV) end-diastolic (LVEDV) and end-systolic (LVESV) volumes, left atrial volume (LAV), and LV ejection fraction (LVEF) were calculated using the modified Simpson’s technique from the apical 2- and 4-chamber images. The LAV index (LAVI) was estimated as a ratio of LAV to BSA. The tricuspid annular plane systolic excursion (TAPSE) and basal right ventricular diameter were detected in the conventional right ventricular (RV)-focused apical four-chamber view obtained with medial transducer orientation [42]. Tissue Doppler recordings and impulse Doppler captures were received from three consecutive beats at the end of expiration from standard apical 2- and 4-chamber views, and average values were used for the final analyses. We evaluated early diastolic blood filling (E), longitudinal strain ratio (e’) and estimated the E/e’ ratio, which was expressed as the ratio equation of E wave velocity to averaged medial and lateral e’ velocities [41]. Left ventricular hypertrophy (LVH) was detected when LV myocardial mass index (LVMMI) was >115 g/m^2^ or >95 g/m^2^ in males and females, respectively [42].

### 2.4. Blood Sampling and Determination of Glomerular Filtration Rate and Insulin Resistance

The collection of blood samples from fasting patients was performed at the same time (from 7:00 to 8:00 a.m.). The blood was collected from an antecubital vein (3–5 mL) and maintained at 4 °C. After centrifugation (3000 r/min, 30 min), polled serum aliquots were immediately stored at ≤−70 °C until analysis. Conventional biochemistry parameters were routinely measured at the local biochemical laboratory of the “Vita-Center” (Zaporozhye, Ukraine) using a Roche P800 analyser (Basel, Switzerland). We used the CKD-EPI formula to estimate the glomerular filtration rate (GFR) [43]. Insulin resistance was evaluated using the Homeostatic Assessment Model of Insulin Resistance (HOMA-IR) [44].

### 2.5. Biomarker Determination

Serum concentrations of NT-proBNP, high-sensitivity C-reactive protein (hs-CRP), and adropin were determined using commercially available enzyme-linked immunosorbent assay (ELISA) kits (Elabscience, Houston, TX, USA) according to the manufacturer’s instructions. All ELISA data were analysed according to the standard curve, and each sample was measured in duplicate as the mean value was finally analysed. Both the intra- and inter-assay coefficients of variability for each biomarker were <10%.

### 2.6. Statistics

Statistical analysis was executed using SPSS 21.0 for Windows (IBM Corp., Armonk, NY, USA) and GraphPad Prism version 9 (GraphPad Software, San Diego, CA, USA). Continuous variables were expressed as means (M) ± standard deviation (SD) or median (Me) and interquartile range [IQR] depending on the presence or absence of a normal distribution, respectively. The Kolmogorov–Smirnov test was used as an assessment for normal distribution. The distribution of dichotomous values was analysed using the chi-square test. Data between two groups were compared with an unpaired *t*-test and a Mann–Whitney U test, while those among multiple groups were assessed by one-way analysis of variance (ANOVA) followed by Tukey’s post hoc test. The Spearman r coefficient was used for correlations between the levels of adropin and other parameters. The Receive Operation Characteristics (ROC) curve analysis was used to assess predictive performance. We detected the optimal cut-off point for adropin with the Jouden index and evaluated the model’s area under the curve (AUC), confidence interval (CI), sensitivity, and specificity. Predictors of CKD were determined by univariate and multivariate logistic regression analysis. We reported the odds ratio (OR) and the 95% confidence interval (95% CI) for each predictor. Differences were considered significant at the level of statistical significance, *p* < 0.05.

## 3. Results

### 3.1. General Characteristics of the Patients

The entire number of T2DM patients with known HF consists of 231 male (55.4%) and 186 female (44.6%) patients with an average age of 53 (41–64) years who have a large spectrum of comorbidities and concomitant diseases, including dyslipidaemia (83.0%), hypertension (84.4%), stable coronary artery disease (33.8%), smoking (40.3%), abdominal obesity (42.9%), left ventricular (LV) hypertrophy (80.1%), atrial fibrillation (13.7%), microalbuminuria (18.0%), and macroalbuminuria/proteinuria (3.8%) (Table 1). The control groups were age- and gender-matched groups of 25 healthy volunteers and 30 T2DM non-HF patients without CKD.

Among the entire number of T2DM HF patients, HFpEF, HFmrEF, and HFrEF were detected in 31.7%, 33.6%, and 34.8% of patients, respectively. We established I/II HF New York Heart Association (NYHA) class in 67.6% of patients; others (32.4%) had III HF NYHA class. All patients were hemodynamically stable and had average values of LVEF of 46 (37–55)%, LVMMI of 154 ± 5 g/m^2^, LAVI of 43 (37–52) mL/m^2^, E/e’ of 13.5 ± 0.3 units, basal RV diameter of 24 (12–36) mm, and TAPSE of 25 (21–28) mm. Fasting levels of creatinine, glucose, and NT-proBNP were 108.6 ± 8.5 µmol/L, 6.12 ± 1.3 mmol/L, and 2615 (1380–3750) pmol/mL, respectively. All HF patients received conventional therapy, depending on their HF phenotype. The majority of them were treated with SGLT2 inhibitors, metformin, and statins. We did not find any significant differences between CKD and non-CKD groups in age, gender, BMI, waist circumference, WHR, a presentation of concomitant diseases and risk factors, apart from albuminuria, proteinuria, and atrial fibrillation (AF), which were detected more frequently in CKD group patients than those with non-CKD. Therefore, there were no significant differences between CKD and non-CKD patients in HF phenotypes, NYHA classes, and main haemodynamic performances, including LVEF, basal RV diameter, TAPSE, as well as the levels of circulating biomarkers. In fact, non-CKD patients had lower E/e’ and more often received mineralocorticoid receptor antagonists when compared with patients from the CKD group.

### 3.2. Circulating Levels of Adropin in T2DM HF Patients with and without CKD Compared with Healthy Volunteers and Non-HF/Non-CKD Diabetics

**Figure 2:**
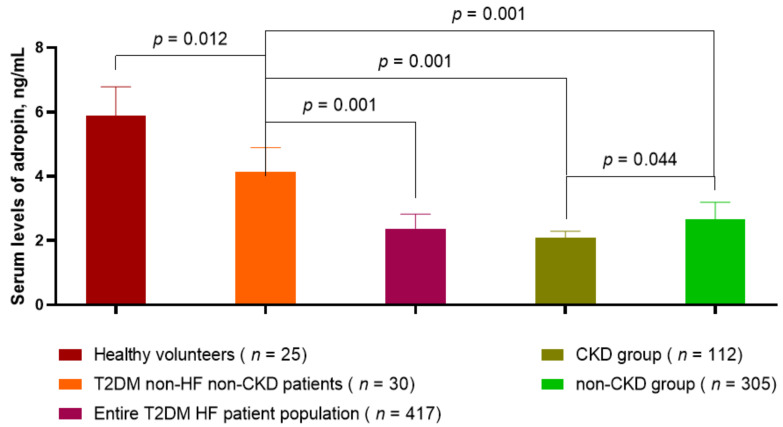
The levels of adropin (ng/mL) in the circulating blood of T2DM patients and healthy volunteers. Abbreviations: CKD, chronic kidney disease; HF, heart failure; T2DM, type 2 diabetes mellitus.

The levels of circulating adropin in T2DM patients without HF and CKD were significantly lower (4.15 ng/mL, 95% confidence interval (CI) = 3.72–4.60 ng/mL) than in healthy volunteers (5.88 ng/mL, 95% CI = 4.90–7.10 ng/mL, *p* = 0.012), but they were higher than in T2DM patients with known HF (2.37 ng/mL, 95% CI = 1.90–2.75 ng/mL, *p* = 0.001) (Figure 2). Therefore, there was a significant difference between adropin levels in T2DM HF patients with and without CKD (2.08 ng/mL, 95% CI = 1.82–2.33 ng/mL, and 2.65 ng/mL, 95% CI = 2.06–3.11 ng/mL, respectively, *p* = 0.001). The levels of adropin in these groups of T2DM HF patients were significantly lower than in healthy volunteers and T2DM without HF and CKD.

### 3.3. Spearman’s Correlation between Circulating Levels of Myokines and Other Parameters

We found that in the entire T2DM HF patient population the levels of adropin were found to be negatively correlated with LVEF (r = −0.58, *p* = 0.001), NYHA class (r = −0.30, *p* = 0.012), BMI (r = −0.29, *p* = 0.012), hs-CRP (r = −0.28, *p* = 0.001), triglycerides (r = −0.23; *p* = 0.044), fasting plasma glucose (r = −0.22; *p* = 0.042), HOMA-IR (r = −0.27; *p* = 0.001), and HbA1c (r = −0.24; *p* = 0.010), while positively correlated with NT-proBNP levels (r = 0.36; *p* = 0.001), LAVI (r = 0.32; *p* = 0.001), high-density lipoprotein cholesterol (r = 0.26; *p* = 0.001), and eGFR (r = 0.30; *p* = 0.001). Aligned with it, adropin levels were significantly associated with HFrEF (r = −0.34; *p* = 0.001) in the CKD group, whereas in the non-CKD group we did not notice such a correlation. There was a positive correlation between the levels of adropin and the use of SGLT2 inhibitors (r = 0.38, *p* = 0.001), whereas other concomitant medications did not exert any significant associations with this parameter. Adropin levels did not correlate with albuminuria and proteinuria.

### 3.4. ROC Curve Analysis of the Predictive Value of Adropin for CKD in T2DM Patients with HF

**Figure 3:**
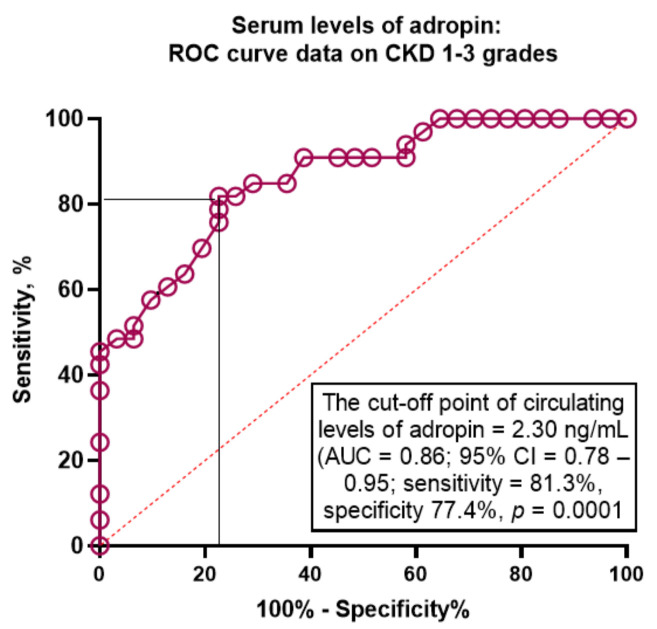
The predictive model based on the serum levels of adropin for CKD 1–3 grades: The results of ROC curve analysis. Abbreviations: AUC, area under the curve; CI, confidence interval; HF, heart failure; CKD, chronic kidney disease; ROC, Receive Operation Curve; T2DM, type 2 diabetes mellitus.

The Receive Operation Curve (ROC) analysis (Figure 3) yielded the optimal cut-off point for serum levels of adropin (versus non-CKD HF T2DM) at 2.3 ng/mL (area under the curve (AUC) = 0.86; 95% CI = 0.78–0.95; sensitivity = 81.3%, specificity = 77.4%; likelihood ratio = 3.623; *p* = 0.0001).

### 3.5. Predictive Models for CKD in T2DM Patients with HF: Univariate and Multivariate Logistic Regression Analysis Adjusted to Albuminuria/Proteinuria

We used univariate logistic regression variables (BMI, age, E/e’, and LAVI), which were structured depending on their median value in the entire T2DM HF population, the cut-off point level of adropin, and the presence versus absence of several conditions, including left ventricular (LV) hypertrophy, atrial fibrillation, and the use of SGLT2 inhibitors (Table 2). The univariate logistic regression adjusted for albuminuria/proteinuria revealed that CKD was predicted by the following variables: serum levels of adropin <2.30 ng/mL (OR = 1.84; *p* = 0.001), LV hypertrophy (OR = 1.10; *p* = 0.022), age ≥ 53 years (OR = 1.05; *p* = 0.044); E/e’ (OR = 1.08; *p* = 0.010), and LAVI ≥ 43 mL/m^2^ (OR = 1.06; *p* = 0.001). The multivariate logistic regression showed that serum levels of adropin < 2.30 ng/mL (OR = 1.55; *p* = 0.001) retained their independence as a predictor for CKD.

## 4. Discussion

The results of our study showed that the circulating levels of adropin < 2.30 ng/mL independently predicted CKD 1–3 grades in T2DM patients with chronic HF. This finding may shed new light on the use of biomarker-guided management in HF patients with metabolic comorbidities, including T2DM. Indeed, the presence of CKD in HF patients sufficiently constrains the proof-of-decision for therapies, while the risk of poor clinical outcomes in CKD patients is reported to be higher than that of those who do not have CKD [39]. There is restrictive evidence of the reproducibility and predictability of conventional kidney injury biomarkers, including albuminuria, proteinuria, albumin/creatinine ratio, eGFR, cystatin C, as well as cardiac biomarkers (natriuretic peptides, cardiac troponins) for CKD in HF patients with concomitant T2DM [45,46]. Thus, our results seem to show novel potency for adropin in a selective group of patients with HF.

Although adropin has been previously investigated in numerous animal and clinical studies as a biomarker of T2DM-induced nephropathy, endothelial dysfunction, arterial stiffening, and atherosclerosis [24,47,48,49], there are scarce studies regarding its predictive ability in the HF population with several concomitant diseases, including T2DM and obesity [50]. Accumulating data suggest that adropin exerts metabolic regulation of gluconeogenesis, ketone production, and lipid oxidation in the myocardium, liver, and skeletal muscle [51,52]. Yet, adropin ameliorates the flexibility of metabolic homeostasis through increasing glycolytic flux via both oxidative and non-oxidative pathways and downregulating skeletal muscle fatty acid uptake by an expression of the sarcolemmal fatty acid translocase [51,53,54]. Finally, adropin improves insulin sensitivity, suppresses oxidative stress and mitochondrial dysfunction, and attenuates the worsening repair potency of precursors via the activation of Akt phosphorylation, transcription 3 (STAT3) signalling, the glucose transporter 4 receptor, and tyrosine protein kinase JAK2 (JAK2)/signal transducer pathways [55,56]. In addition to that, adropin was able to directly promote microangiogenesis, increase microvessel density, suppress oxidative stress, and inhibit myocardial fibrosis and apoptosis regardless of its capability of ameliorating glucose and lipid metabolism [57]. These effects are promoted by adropin through downregulation of the expression levels of transforming growth factor *β*1, NADPH oxidase 4, and cleaved caspase 3, and upregulation of the expression of phosphor-endothelial nitric oxide synthase [57].

In a clinical context, all these mean that adropin exerts tissue protective capability via numerous distinguished mechanisms and that a decreased pool of this circulating peptide is considered a powerful marker of CV risk. Indeed, low levels of adropin were found in patients with arterial hypertension and atherosclerosis [22,58,59,60]. Moreover, patients with overweight/obesity and known T2DM and CKD exhibited lower levels of adropin when compared with healthy volunteers [23,61,62,63]. Yet, low levels of adropin were found in patients with acute myocardial infarction [64] and stable coronary artery disease (CAD) [65]. In fact, decreased circulating concentrations of adropin in peripheral blood corresponded to a higher risk of T2DM, CAD, and CKD, but not for HF [66].

The results of our study showed that the levels of adropin in chronic HF patients with T2DM were lower than in healthy volunteers and T2DN individuals without HF, whereas in other studies [31,66] elevated levels of adropin were detected in HF patients. We, however, suggested that the use of adropin could be practically useful in patients with T2DM and HF regardless of CKD to stratify the patients at risk of CKD and provide continuous monitoring for risk modification during treatment, including a prediction of target organ damages and a response to the therapy. Perhaps our findings will result in the implementation of a guideline-recommended HF treatment programme. Indeed, all individuals were clinically stable and treated with optimal combinations, including SGLT2 inhibitors, renin-angiotensin-aldosterone antagonists, and beta-blockers. The main causes of changeable levels of adropin in HF might be a suppression of adropin synthesis and release due to inflammatory and neurohumoral activation in connection with concomitant circulatory deficiency and subsequent tissue ischemia/hypoxia, poor kidney clearance of adropin, declining skeletal muscle mass and adipose mass tissue, malabsorption and failed digestion of nutrients, and psychological problems [67]. It is reasonable to suggest that the dynamics of the adropin level might depend on the complex interplay of these factors. Indeed, recently Xu W et al. (2021) [30] reported that the levels of adropin were found to be increased during effective treatment of chronic HF, whereas others noticed that pre-existing increased levels of the peptide were strongly associated with the severity of HF [31,65]. However, all investigators confirmed that the concentrations of adropin correlated with BMI, NT-proBNP, the lipid profile, HOMA-IR, and biomarkers of inflammation such as hs-CRP, interleukin-6, and LVEF. However, positive or negative relations between adropin levels and other parameters are still controversial. We established that adropin levels were negatively associated with LVEF, NYHA class, BMI, hs-CRP, and some parameters of glucose (fasting plasma glucose, HOMA-IR, and HbA1c) and lipid metabolism (serum levels of triglycerides), and positively correlated with NT-proBNP levels, LAVI, high-density lipoprotein cholesterol, and eGFR. In fact, these factors are considered plausible variables for univariate logistic analysis to verify possible predictors for CKD. We adjusted the analysis to account for albuminuria/proteinuria and found that adropin independently predicted CKD in HF patients with T2DM.

**Figure 4:**
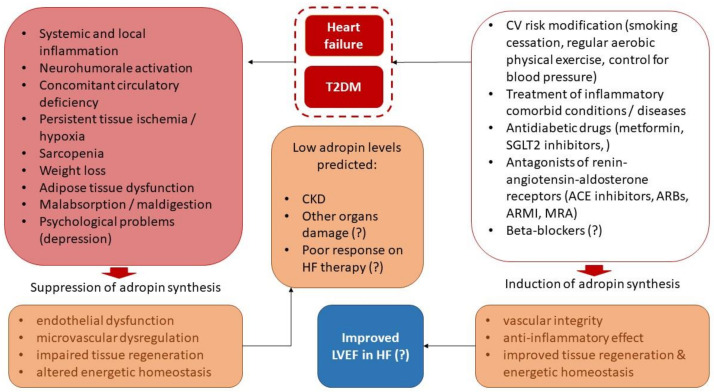
The plausible diagnostic and predictive values of adropin in patients with HF and concomitant T2DM. Abbreviations: ARBs, angiotensin-II receptor blockers; ARNI, angiotensin receptor neprilysin inhibitor; HF, heart failure; ACE, angiotensin-converting enzyme; CKD, chronic kidney disease; LVEF, left ventricular ejection fraction; SGLT2, sodium-glucose co-transporter 2; T2DM, type 2 diabetes mellitus; ?, no strong evidence.

One possible explanation of the role of adropin in the prediction of CKD is its close relationship to the severity of endothelial dysfunction and microvascular dysregulation, which have been previously disputed as triggers of kidney injury resulting in CKD [68]. Perhaps the concept of the kidney vascular endothelium playing a key role in supporting effective kidney perfusion in HF is more promising to explain the restrictive tissue protective impact of low levels of adropin on this target organ. The next explanation is based on the idea of a presence of persistence injury to tubular epithelial cells in the kidney during the natural evolution of HF [69]. The metabolic capabilities of adropin seem to show a protective influence on tubular apparatus regardless of fluctuations in eGFR. Thus, persistent ischemia in the kidney may be ameliorated by increased production of adropin, so that elevated levels of the peptide might be a marker of adaptive mechanisms, by which a risk of target organ damage is diminished. On the contrary, low levels of adropin characterise a maladaptive shift in energy homeostasis due to overexpression of pro-inflammatory genes and overproduction of inflammatory cytokines, which are supported by T2DM-induced oxidative stress and mitochondrial dysfunction [69]. Plausible diagnostic and predictive values of adropin are illustrated in Figure 4.

Whether a low level of adropin retains its discriminative value for CKD in HF patients with T2DM depending on HF phenotype, nutrient status, the spectrum of comorbidities, and fluid retention/overload is under scientific discussion and requires more investigations in the future.

## 5. Study Limitations

This study has several limitations. The first is the small number of healthy volunteers and T2DM patients without CKD and HF. The secondary limitation relates to a lack of nutrient status data for the patients, while we did not change their diet we recommended enhancing their optimal food plan. At last, but not least, we did not investigate a link between changes in adipose tissue mass/skeletal muscle mass and ketogenesis in the context of the possible impact of these processes on adropin. Therefore, we did not have clear evidence of kidney perfusion. We believe these ideas may be taken into consideration for planning new investigations in the future. Therefore, we think these limitations will not interfere with the acceptance of our hypothesis and data interpretation.

## 6. Conclusions

We established that circulating levels of adropin in HF patients with T2DM were significantly lower than those in healthy volunteers and T2DM individuals without HF. Moreover, adropin levels < 2.30 ng/mL independently predicted CKD 1–3 grades in T2DM patients with chronic HF, which may be considered a novel biomarker-based approach for stratifying HF patients at CKD risk during management.

## Figures and Tables

**Table 1:** Baseline general characteristics of eligible T2DM patients compared with healthy volunteers and T2DM non-HF patients.

Variables	Healthy Volunteers (*n* = 25)	T2DM Non-HF Patients without CKD (*n* = 30)	T2DM HF Patients			
			Entire Patient Cohort (*n* = 417)	CKD Patients (*n* = 112)	Non-CKD Patients (*n* = 305)	*p* Value
Demographics and anthropomorphic parameters						
Age, year	51 (47–55)	52 (47–55)	53 (41–64)	56 (44–67)	52 (40–65)	0.12
Male/female *n* (%)	14 (56.0)/11 (44.0)	17 (57.0)/13 (43.0)	231 (55.4)/186 (44.6)	62 (55.3)/50 (44.7)	169 (55.4)/136 (44.6)	0.90
BMI, kg/m^2^	23.9 ± 2.7	24.1 ± 2.3	25.8 ± 2.8	25.0 ± 3.2	26.2 ± 2.3	0.66
Waist circumference, cm	86.1 ± 3.6	86.1 ± 3.6	95.1 ± 3.2 **	94.7 ± 3.0	96.2 ± 4.1	0.58
WHR, units	0.73 ± 0.04	0.84 ± 0.05 *	0.85 ± 0.05	0.84 ± 0.06	0.87 ± 0.09	0.80
Concomitant diseases and risk factors						
Dyslipidaemia, *n* (%)	-	24 (80.0)	346 (83.0)	96 (85.7)	250 (81.9)	0.14
Hypertension, *n* (%)	-	13 (43.3)	352 (84.4) **	105 (93.8)	247 (81.0)	0.04
Stable CAD, *n* (%)	-	-	141 (33.8) **	43 (38.4)	98 (32.1)	0.05
Smoking, *n* (%)	4 (16)	11 (36.7) *	168 (40.3)	41 (36.6)	127 (41.6)	0.06
Abdominal obesity, *n* (%)	-	9 (30.0) *	179 (42.9) **	45 (40.2)	134 (43.9)	0.22
LV hypertrophy, *n* (%)	-	7 (23.3) *	334 (80.1) **	93 (83.1)	241 (79.0)	0.042
AF, *n* (%)	-	-	57 (13.7) **	21 (18.7)	36 (11.8)	0.04
Albuminuria, *n* (%)	-	-	75 (18.0) **	47 (42.0)	28 (9.2)	0.001
Proteinuria, *n* (%)	-	-	16 (3.8) **	16 (9.8)	-	0.001
HF phenotypes and functional classification						
HFpEF, *n* (%)	-	-	132 (31.7) **	32 (28.6)	100 (32.3)	0.10
HFmrEF, *n* (%)	-	-	140 (33.6) **	39 (34.8)	101 (33.1)	0.14
HFrEF, *n* (%)	-	-	145 (34.8) **	41 (36.6)	103 (33.8)	0.12
I/II HF NYHA class, *n* (%)	-	-	282 (67.6) **	68 (60.7)	214 (70.1)	0.04
III HF NYHA class, *n* (%)	-	-	135 (32.4) **	44 (39.3)	91 (29.9)	0.04
Haemodynamic features						
SBP, mm Hg	124 ± 5	129 ± 7	132 ± 7	135 ± 6	130 ± 6	0.22
DBP, mm Hg	73 ± 4	76 ± 5	78 ± 5	79 ± 4	76 ± 5	0.20
LVEDV, mL	141 (122–155)	144 (126–158)	162 (154–170) **	165 (157–174)	160 (153–168)	0.05
LVESV, mL	52 (44–61)	55 (47–64)	86 (80–93) **	91 (82–97)	85 (80–91)	0.05
LVEF, %	63 (59–68)	62 (56–67)	46 (37–55) **	45 (35–56)	46 (37–57)	0.64
LVMMI, g/m^2^	102 ± 4	108 ± 5	154 ± 5 **	156 ± 4	151 ± 7	0.48
LAVI, mL/m^2^	31 (29–34)	34 (30–36)	43 (37–52) **	45 (38–50)	42 (35–50)	0.14
E/e’, unit	6.45 ± 0.3	6.51 ± 0.4	13.5 ± 0.3 **	15.1 ± 0.6	11.4 ± 0.5	0.001
TAPSE, mm	25 (21–28)	24 (20–28)	21 (18–25)	19 (16–23)	22 (20–26)	0.052
basal RV diameter, mm	24 (12–36)	26 (13–40)	28 (15–43)	29 (17–45)	25 (15–42)	0.050
Biomarkers						
eGFR, mL/min/1.73 m^2^	109 ± 6	109 ± 7	75 ± 18 **	63 ± 12	96 ± 6	0.02
HOMA-IR	4.32 ± 0.7	5.95 ± 0.9 *	7.95 ± 2.3	8.60 ± 2.0	6.90 ± 2.2	0.40
Fasting glucose, mmol/L	5.1 ± 0.7	6.08 ± 0.8 *	6.12 ± 1.3	6.20 ± 1.5	5.80 ± 1.6	0.80
HbA1c, %	5.20 ± 0.04	6.40 ± 0.05 *	6.59 ± 0.02	6.71 ± 0.09	6.50 ± 0.03	0.68
Creatinine, µmol/L	69.5 ± 7.0	77.4 ± 8.0	108.6 ± 8.5 **	119.2 ± 5.5	96.3 ± 4.7	0.01
TC, mmol/L	4.93 ± 0.50	5.48 ± 0.40 *	6.43 ± 0.60 **	6.50 ± 0.50	6.40 ± 0.40	0.78
HDL-C, mmol/L	1.04 ± 0.12	1.01 ± 0.15	0.97 ± 0.17	0.96 ± 0.16	0.99 ± 0.19	0.80
LDL-C, mmol/L	2.88 ± 0.13	3.10 ± 0.14 *	4.38 ± 0.10 **	4.43 ± 0.13	4.30 ± 0.14	0.82
TG, mmol/L	1.70 ± 0.10	1.80 ± 0.12	2.21 ± 0.17 **	2.21 ± 0.14	2.22 ± 0.16	0.88
hs-CRP, mg/L	0.75 (0.32–1.10)	4.03 (2.90–5.36) *	7.12 (4.33–10.51)	8.45 (5.12–11.30)	6.24 (4.07–8.87)	0.05
NT-proBNP, pmol/mL	48 (10–95)	56 (0–102)	2615 (1380–3750) **	2844 (1560–3810)	2598 (1280–3880)	0.46
Concomitant medications						
ACEI, *n* (%)	-	13 (43.3) *	198 (47.5)	52 (46.4)	146 (47.9)	0.78
ARB, *n* (%)	-	-	67 (16.1) **	27 (24.1)	40 (13.1)	0.02
ARNI, *n* (%)	-	-	165 (39.6) **	41 (36.6)	124 (40.6)	0.11
Beta-blocker, *n* (%)	-	-	372 (89.2) **	103 (92.0)	269 (88.2)	0.44
Ivabradine, *n* (%)	-	-	59 (14.1) **	12 (10.7)	47 (15.4)	0.05
Calcium channel blocker, *n* (%)	-	5 (16.7) *	75 (18.0)	22 (19.6)	53 (17.3)	0.06
MRA, *n* (%)	-	-	283 (67.8) **	46 (41.1)	237 (77.7)	0.01
SGLT2 inhibitor, *n* (%)	-	12 (40.0) *	362 (86.8) **	92 (82.1)	270 (88.5)	0.20
Loop diuretic, *n* (%)	-	-	358 (85.9) **	110 (98.2)	248 (81.3)	0.04
Antiplatelet, *n* (%)	-	-	367 (88.0) **	98 (87.5)	269 (88.2)	0.86
Anticoagulant, *n* (%)	-	-	57 (13.7) **	21 (18.7)	36 (11.8)	0.04
Metformin, *n* (%)	-	30 (100) *	387 (92.8)	86 (76.8)	301 (98.7)	0.04
Statins, *n* (%)	-	24 (80.0) *	408 (97.8)	103 (92.0)	305 (100)	0.05

Notes: data of variables are given as mean ± SD and median (25–75% interquartile range), *p* value, a difference between values in CKD and non-CKD patients, CKD is referred to as grades 1–3, *, statistical difference (*p* < 0.05) between healthy volunteers and T2DM non-HF patients; **, statistical difference (*p* < 0.05) between T2DM non-HF patients and T2DM HF patients. Abbreviations: ACEI, angiotensin-converting enzyme inhibitor; ARBs, angiotensin-II receptor blockers; ARNI, angiotensin receptor neprilysin inhibitor; CAD, coronary artery disease; CKD, chronic kidney disease; BMI, body mass index; DBP, diastolic blood pressure; E/e’, early diastolic blood filling to the longitudinal strain ratio; eGFR, estimated glomerular filtration rate; hs-CRP, high-sensitivity C-reactive protein; HDL-C, high-density lipoprotein cholesterol; HFpEF, heart failure with preserved ejection fraction; HFmrEF, heart failure with mildly reduced ejection fraction; HFrEF, heart failure with reduced ejection fraction; LVEDV, left ventricular end-diastolic volume; LVESV, left ventricular end-systolic volume; LVEF, left ventricular ejection fraction; LVMMI, left ventricle myocardial mass index, left atrial volume index, LAVI; left atrial volume index; LDL-C, low-density lipoprotein cholesterol; MRA, mineralocorticoid receptor antagonist; SBP, systolic blood pressure; RV, right ventricle; TAPSE, tricuspid annular plane systolic excursion; TG, triglycerides; TC, total cholesterol; WHR, waist-to-hip ratio.

**Table 2:** Predictors for CKD in the T2DM HF population. The results of the univariate and multivariate logistic regression analyses were adjusted for albuminuria/proteinuria.

Variables	Depending Variables: CKD 1–3 Grades					
	Univariate Log Regression		Multivariate Log Regression			
	OR	95% CI	*p*-Value	OR	95% CI	*p*-Value
Adropin < 2.30 ng/mL vs. ≥2.30 ng/mL	1.84	1.34–2.41	0.001	1.55	1.38–1.86	0.001
LVH vs. non LVH	1.10	1.07–1.15	0.022	1.06	1.00–1.13	0.16
BMI < 25.8 кг/м^2^ vs. ≥25.8 кг/м^2^	1.06	1.00–1.13	0.052	-		
Age ≥ 53 years vs. <53 years	1.05	1.01–1.12	0.044	1.03	1.00–1.05	0.46
Smoking vs. non-smoking	1.02	0.93–1.12	0.88	-		
E/e’ > 13.5 vs. ≥13.5 units	1.08	1.03–1.14	0.010	1.04	1.00–1.09	0.44
LAVI ≥ 43 mL/m^2^ vs. <43 mL/m^2^	1.06	1.01–1.10	0.001	1.05	0.97–1.09	0.62
Use of SGLT2i vs. unuse of SGLT2i	0.92	0.87–1.01	0.72	-		
AF vs. non-AF	1.10	0.88–1.24	0.86	-		

Abbreviations: BMI, body mass index; CI, confidence interval; LV, left ventricle; LAVI, left atrial volume index; E/e’, early diastolic blood filling to longitudinal strain ratio; SGLT2i, sodium-glucose co-transporter 2-inhibitors; OR, odds ratio.

## Data Availability

Not applicable.
